# Traumatic Cervical Myelopathy Masked by Alcohol Intoxication and Diagnostic Anchoring

**DOI:** 10.7759/cureus.102868

**Published:** 2026-02-03

**Authors:** Abraham Gabriel, Claudia Gabriel, Maria Georgi, Michael Milad, Monica Kelada

**Affiliations:** 1 Ophthalmology, Ashford and St. Peter's Hospitals NHS Foundation Trust, Chertsey, GBR; 2 Otolaryngology-Head and Neck Surgery, Kings College Hospital, London, GBR; 3 Ophthalmology, Chelsea and Westminster Hospital NHS Foundation Trust, London, GBR; 4 Oncology, West Hertfordshire NHS Trust, Watford, GBR; 5 Medicine, Imperial College Healthcare NHS Trust, London, GBR

**Keywords:** acute hyperextension-induced myelopathy, autonomic nervous system dysfunction, compression neuropathy, ethanol intoxication, hyperextension of the neck, intensive care and invasive monitoring, lower limb paralysis, mri in spinal trauma, spinal cord injury, upper limb paralysis

## Abstract

Acute alcohol intoxication often masks traumatic injuries, leading to diagnostic anchoring and delayed management. We report the case of a 61-year-old male presenting with sudden-onset quadriplegia. Initial clinical suspicion favoured a cerebrovascular accident or Guillain-Barré syndrome due to the patient’s amnesia about a fall and the presence of new-onset atrial fibrillation. A significant diagnostic delay occurred until collateral history from peers revealed a “faceplant” injury, and the retrospective identification of a cutaneous imprint from the patient’s eyeglasses confirmed a hyperextension mechanism. Despite an initial report of a fracture, subsequent imaging confirmed an isolated, severe cord contusion from C3 to C6 without associated bony disruption. This report highlights the importance of the fundamentals of the diagnostic process, including comprehensive history gathering and a thorough bedside examination.

## Introduction

Traumatic spinal cord injury (TSCI) is a devastating event that carries a high burden of permanent disability, with cervical injuries being the most common and morbid [1]. In the aging population, degenerative changes such as cervical spondylosis and canal stenosis significantly increase the vulnerability of the spinal cord to acute traumatic myelopathy, often following low-energy hyperextension mechanisms. A particularly challenging clinical entity is spinal cord injury without radiographic abnormality, which in adults typically involves central cord syndrome or extensive contusion despite negative CT findings [2]. The clinical assessment of TSCI is frequently confounded by acute alcohol intoxication, which serves as a major barrier to accurate history taking and physical examination in the emergency department (ED). Alcohol intoxication not only contributes to the mechanism of injury but also masks pain and induces amnesia about the event, often leading clinicians toward medical diagnostic pathways [3].

The absence of a reliable trauma history in an intoxicated patient shifts clinical priority toward a medical differential diagnosis, where acute neurological deficits are often misattributed to medical pathologies rather than occult spinal trauma. This diagnostic shift can lead to the omission of spinal precautions and delays in critical care interventions, such as hemodynamic optimisation, which are vital for preventing secondary cord injury [4]. Prevalence data suggest that alcohol is a factor in up to 25% of all TSCI admissions, yet its role as a diagnostic distractor remains underreported [5]. Delayed diagnosis in these scenarios is associated with poor functional outcomes and prolonged hospitalisation. This case report is of critical importance as it highlights the need to maintain a high index of suspicion for trauma when neurological deficits are disproportionate to radiologic findings. It demonstrates how attention to subtle cutaneous signs, such as a facial imprint from glasses, can redirect the diagnostic approach and ensure appropriate stabilisation.

## Case presentation

A 61-year-old male with a history of hypertension and type 2 diabetes was brought to the ED by ambulance with acute dense quadriplegia following a night of heavy alcohol consumption. Upon initial examination, the patient was alert but amnestic regarding events from the prior evening. He demonstrated 0/5 MRC (Medical Research Council) power in the upper limbs and minimal movement in the lower limbs (1/5 MRC). Cranial nerve examination was unremarkable, with bilateral 3 mm pupil diameters, equally reactive to light, and no relative afferent pupillary defect. He demonstrated hyperreflexia in the upper and lower limbs, with plantar reflexes upgoing bilaterally (Babinski reflexes). Electrocardiography demonstrated new-onset atrial fibrillation. Given the clinical context and the patient’s denial of trauma, as corroborated by accompanying family members, the team initially focused on a thromboembolic stroke. He underwent a CT scan of the head, which demonstrated no acute pathology. Following this negative evaluation, acute inflammatory polyneuropathies were considered, specifically Guillain-Barré syndrome.

**Figure 1:**
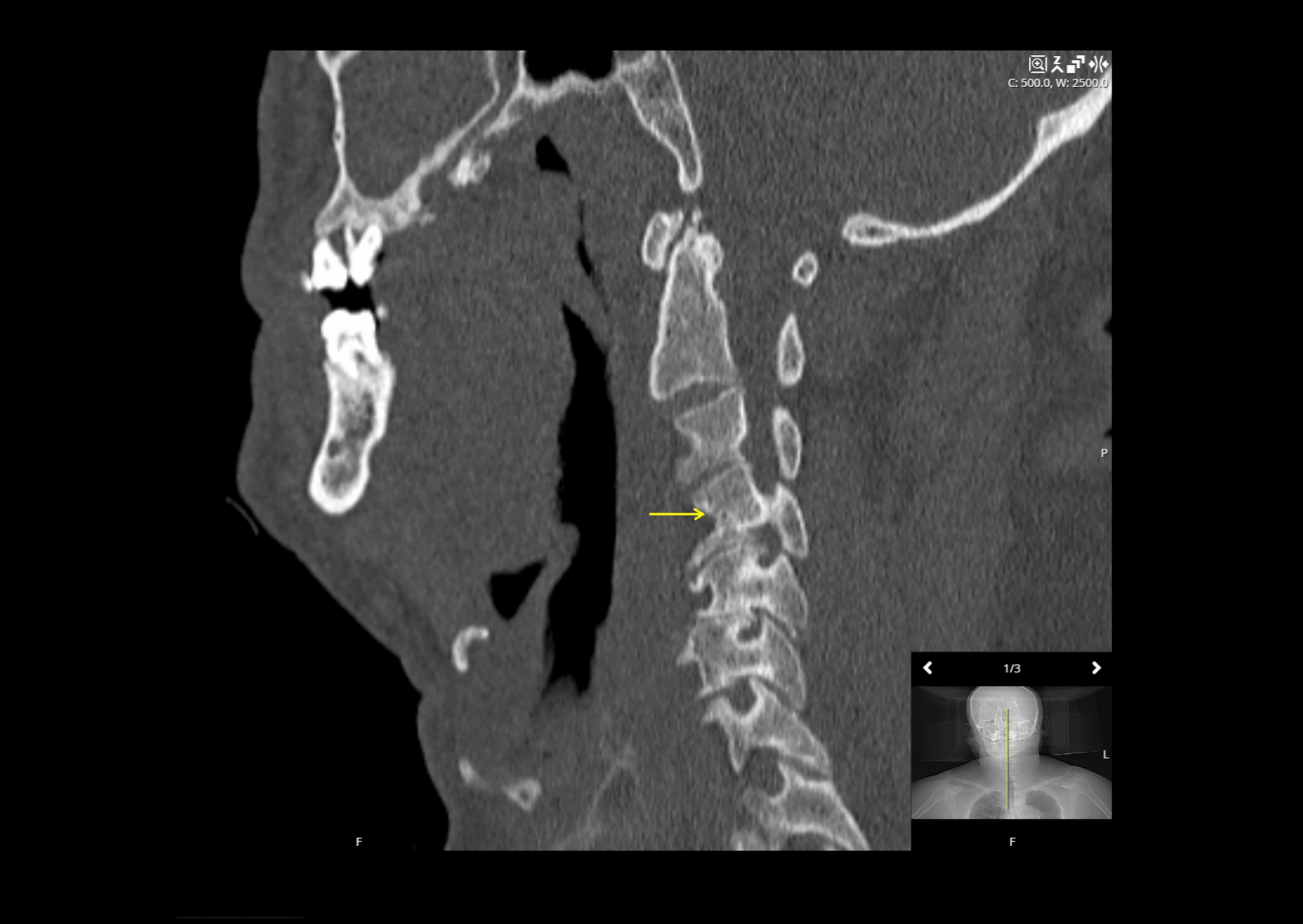
Sagittal CT of the cervical spine The preliminary report suggested a mildly displaced acute fracture involving the anterior inferior endplate of C4 (indicated by the yellow arrow). Subsequent formal review amended this to show chronic spondylotic changes without evidence of acute bony disruption CT: computed tomography

The patient was admitted to a medical ward for further workup; notably, no cervical stabilisation was performed at this time. On the third day of admission, collateral history was obtained from the patient’s acquaintances, who reported witnessing a forward faceplant fall. Following this disclosure, a more meticulous physical examination revealed an indurated, linear imprint across the bridge of the nose and the periorbital region-a cutaneous signature of the patient’s eyeglasses being driven into the face upon impact. As a result of the new trauma history, a CT of the cervical spine was performed. While a preliminary report suggested a C4 endplate fracture (Figure 1), formal review by senior neuroradiologists confirmed the absence of any acute fracture.

**Figure 2:**
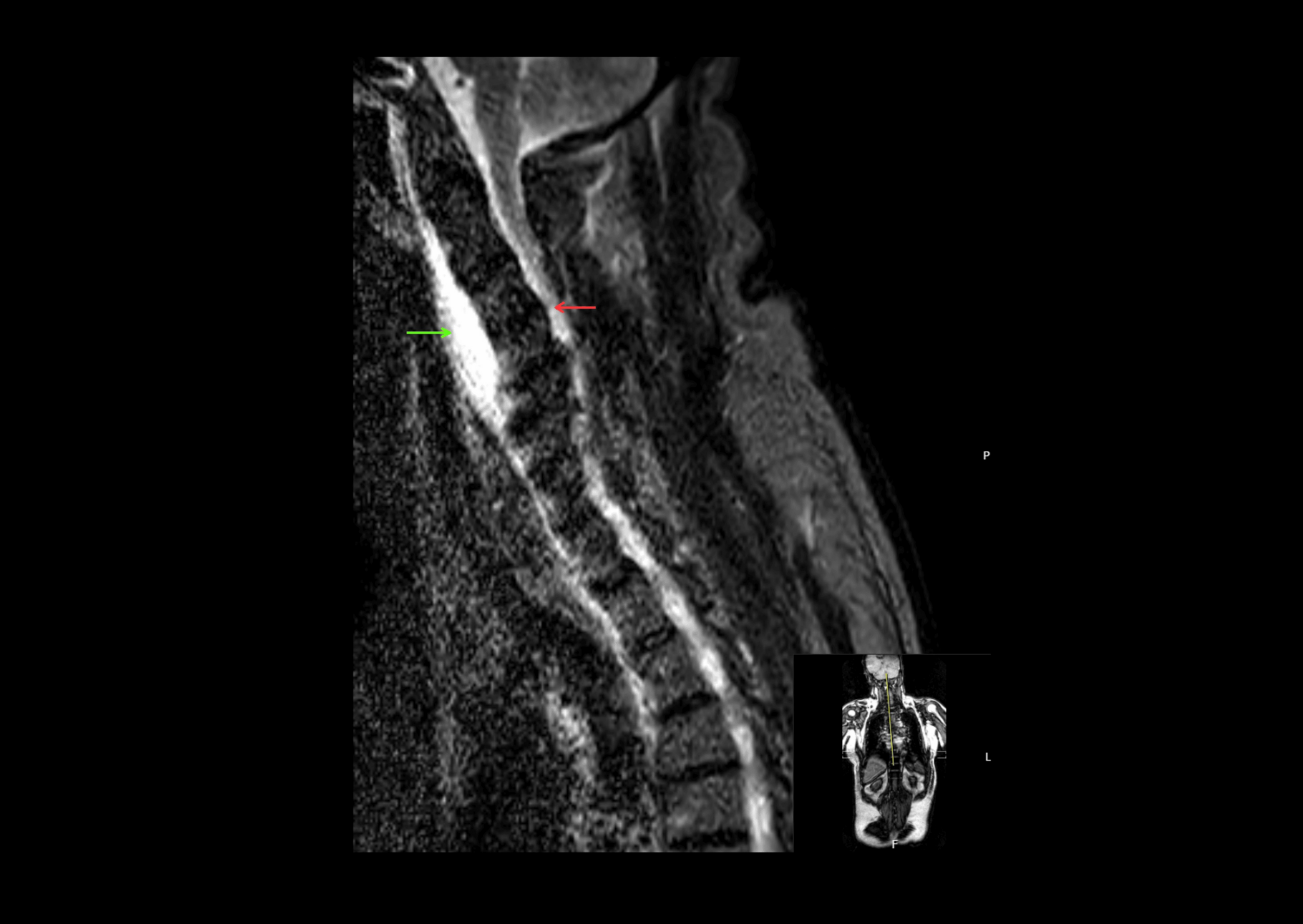
Sagittal T2-weighted MRI of the cervical spine The image demonstrates significant multilevel cervical spondylosis with loss of the normal cervical lordosis and associated spinal canal stenosis, characteristic of a central cord predominant injury pattern. The green arrow indicates a long-segment, poorly demarcated area of intramedullary T2-hyperintensity extending from the C3 to C6 vertebral levels, consistent with severe acute traumatic cord contusion and significant vasogenic edema. The red arrow points to a localized region of hyperintense signal within the posterior ligamentous complex and paraspinal soft tissues between the C3 to C6 vertebral levels; this finding is indicative of ligamentous strain or soft tissue signature typical of a high-energy hyperextension mechanism of injury MRI: magnetic resonance imaging

**Figure 3:**
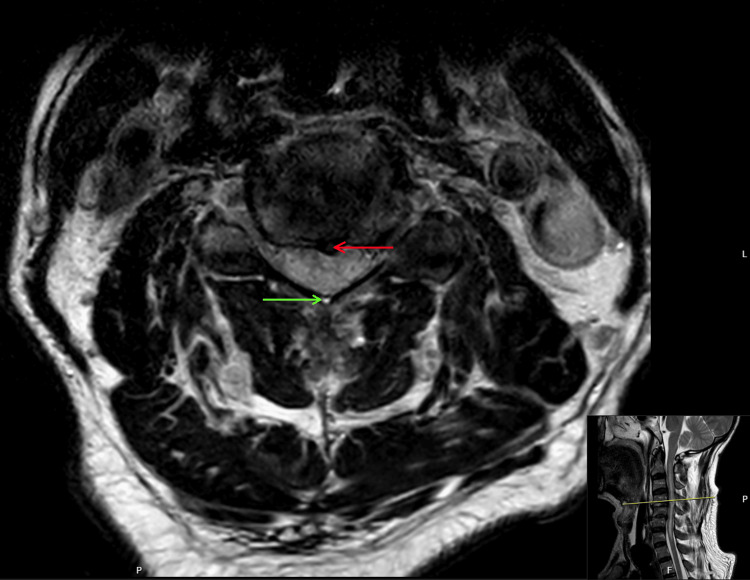
Axial T2-weighted MRI of the cervical spine at the C3 vertebral level An axial cross-section obtained at the level of the C3 vertebral body demonstrates severe mechanical compromise and narrowing of the spinal canal. The green arrow identifies a prominent focus of intramedullary T2-hyperintensity within the substance of the spinal cord, representing acute traumatic contusion and associated vasogenic edema. The spinal cord is visibly deformed and compressed into a characteristic triangular or flattened morphology: a result of the pincer effect between anterior spondylotic osteophytes and the posterior ligamentous complex. This view confirms the localised nature of the neural injury and the significant reduction in the effective canal diameter at this level. This morphology carries a poor functional prognosis MRI: magnetic resonance imaging

MRI of the whole spine was definitive, revealing severe cord compression at the C3-C5 levels. T2-weighted sequences demonstrated extensive intramedullary hyperintensity from C3 to C6, consistent with a severe traumatic cord contusion (Figures 2, 3).

The patient’s condition necessitated ICU admission for respiratory failure and management of profound autonomic dysregulation, characterised by labile blood pressure requiring vasopressor support (noradrenaline) to maintain a mean arterial pressure (MAP) target of 80 mmHg [6], and he was transferred on day four of admission. By day 30, the patient showed signs of neurological improvement, specifically the return of pain sensation in all four limbs-a vital clinical marker of spinothalamic recovery [7]. Following insertion of a percutaneous endoscopic gastrostomy (PEG) for nutritional support, he was transferred to a tertiary neuro-rehabilitation centre for further care.

## Discussion

The alcohol-induced amnesia led to diagnostic anchoring and delay in treatment administration in our patient. Availability bias may have made a stroke diagnosis feel more accessible given the patient's comorbidities, and the subsequent failures of not re-evaluating the trauma history despite atypical clinical features reflect premature closure, or a form of confirmational bias. The initial presentation of quadriplegia and new-onset atrial fibrillation strongly favoured a thromboembolic stroke. Frustratingly, the atrial fibrillation was likely an incidental finding or a physiological reaction to the spinal insult, yet it acted as a significant red herring that further directed the diagnostic focus away from trauma. The etiology of injury in patients with pre-existing cervical spondylosis who experience a hyperextension injury often involves the pincer effect, wherein the cord is compressed between anterior osteophytes and a thickened ligamentum flavum [8]. This can cause devastating cord contusion even when CT imaging is negative for fracture, as seen here. 

Studies by Davis et al. emphasise that missed cervical injuries often involve patient intoxication and a distracted clinical focus [9]. Furthermore, guidelines for acute spinal cord injury management recommend maintaining a MAP between 85 and 90 mmHg for the first seven days to optimise cord perfusion and mitigate secondary ischemic injury [6,10]. In this case, the 72-hour delay in diagnosis postponed optimisation of these critical hemodynamic targets. However, the subsequent return of nociception in this patient is an encouraging sign; historical cohorts show that early sensory recovery, even if limited to pain, correlates with improved long-term motor potential [11]. The lack of a thorough collateral history was the key shortcoming in this patient’s care; ED physicians ought to have contacted the family, who could have redirected the team to the accompanying friends from the excursion, thereby preventing delays. The further failure to identify the cutaneous eyeglass imprint in the ED is another vital learning point; cutaneous markers of head or facial impact should always prompt immediate cervical spine evaluation in the immobile patient, particularly given the lack of history [12].

## Conclusions

Traumatic cervical myelopathy in intoxicated patients presents a significant risk for diagnostic anchoring toward medical mimics. This report emphasises that the absence of bony fractures on CT does not exclude severe cord contusion, particularly in the spondylotic spine. Meticulous physical examination and the persistent pursuit of collateral history are essential to avoid delays in stabilisation and treatment. Early MRI remains the gold standard for defining neural injury when clinical findings and initial imaging are discordant. However, clinicians ought not forgo the fundamentals of history-taking and bedside examination, particularly when there is discordance between radiological evidence and clinical presentation; perhaps a more common occurrence in the investigation-heavy era of modern medical practice.
